# From Spectra to Structure: AI-Powered ^31^P NMR Interpretation

**DOI:** 10.1021/acs.analchem.5c01460

**Published:** 2025-07-16

**Authors:** Marvin Alberts, Nina Hartrampf, Teodoro Laino

**Affiliations:** † 54174 IBM Research Europe, Säumerstrasse 4, 8803 Rüschlikon, Switzerland; ‡ 27217 University of Zurich, Department of Chemistry, Winterthurerstrasse 190, 8057 Zurich, Switzerland; ¶ NCCR Catalysis, Wildhainweg 3, 3001 Bern, Switzerland

## Abstract

Phosphorus-31 nuclear magnetic resonance (^31^P NMR) spectroscopy is a powerful technique for characterizing phosphorus-containing compounds in diverse chemical environments. However, spectral interpretation remains a time-consuming and expertise-dependent task, relying on reference tables and empirical comparisons. In this study, we introduce a data-driven approach that automates ^31^P NMR spectral analysis, providing rapid and accurate predictions of the local phosphorus environments. By leveraging a curated data set of experimental and synthetic spectra, our model achieves a Top-1 accuracy of 53.64% and a Top-5 accuracy of 77.69% at predicting the local environment around a phosphorus atom. Furthermore, it demonstrates robustness across different solvent conditions and outperforms expert chemists by 25% in spectral assignment tasks. The models, data sets, and architecture are openly available, facilitating seamless adoption in chemical laboratories engaged in structure elucidation, with the goal of advancing ^31^P NMR spectral analysis and interpretation.

## Graphical abstract

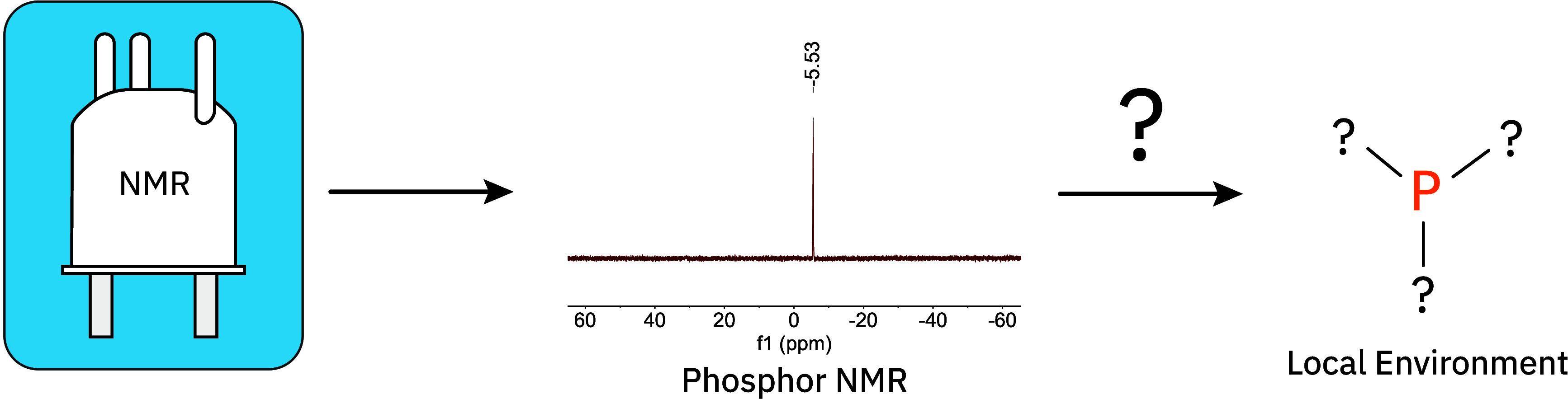

## Introduction

Nuclear magnetic resonance (NMR) spectroscopy is one of the most powerful analytical tools in modern chemistry, offering unparalleled insights into the structure and composition of molecules.^1–3^ Among the various types of spectroscopy, ^31^P NMR spectroscopy is particularly applicable to biological systems, organometallic compounds, and catalysts.^4–6^ The technique’s ability to probe the local environment around phosphorus atoms makes it valuable for structure elucidation as well as quality control in both industrial and academic settings.^7,8^

Despite its broad utility, interpreting ^31^P NMR spectra remains a complex and time-intensive task, requiring expertise and extensive reference data. Assigning peaks typically involves consulting spectral databases or reference tables,^1^ a manual process that is both laborious and susceptible to human error, particularly when dealing with complex molecular structures. Additionally, phosphorus chemical shifts are sensitive to external factors such as solvent choice, temperature fluctuations, and sample concentration, further complicating spectral analysis and often necessitating case-by-case adjustments.^9^

Artificial intelligence (AI) presents an opportunity to overcome these challenges in ^31^P NMR interpretation to facilitate more efficient and reliable spectral analysis. While AI has already demonstrated success in various chemical applications, such as reaction prediction,^10^ retrosynthesis planning,^11,12^ homogeneous^13^ and heterogeneous catalysis,^14^ optimization of reaction processes,^15^ drug design,^16,17^ structure elucidation from IR spectra^18^ and ^1^H and ^13^C NMR spectra,^19^ its potential for ^31^P NMR structure elucidation remains largely unexplored. Recent advances in deep learning, particularly transformer-based models, offer a promising path for automating spectral interpretation by learning patterns from large data sets, as we already demonstrated in the successful use of AI to predict molecular structures from infrared (IR) and ^1^H and ^13^C NMR spectra.^18–20^ A robust AI-driven system could streamline phosphorus spectral analysis, reducing the reliance on manual reference comparisons and improving the accuracy and efficiency of structural assignments.

In this study, we present what is, to the best of our knowledge, the first successful AI-driven approach for predicting the local environment around phosphorus atoms from ^31^P NMR spectra (Figure 1). To achieve this, we generated a large pretraining data set of 665.844 synthetic ^31^P NMR spectra and trained a transformer-based model to predict the local environment around the phosphorus atoms. Additionally, we leveraged the experimental data set published by Hack et al.^21^ integrating real-world spectra to refine the model’s predictive capabilities. Our model achieves a Top-1 accuracy of 53.64% and a Top-5 accuracy of 77.69%, demonstrating consistent performance across different solvent conditions. Furthermore, in direct comparisons, our AI model outperforms human chemists by 25% in spectral assignment, highlighting its potential to enhance and accelerate structure elucidation workflows in both research and industrial laboratories.

**Figure 1:**
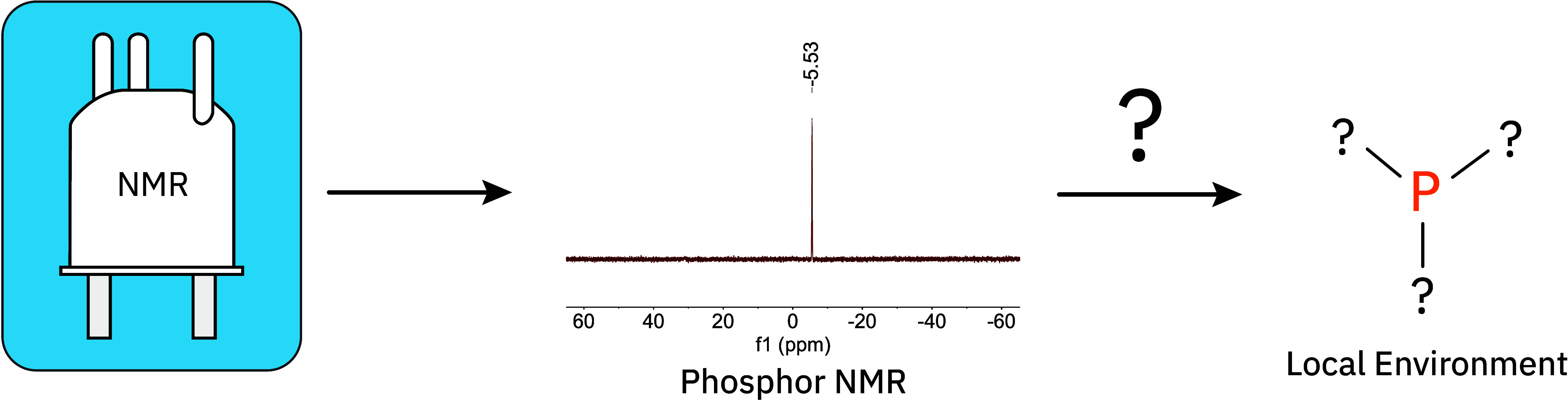
Workflow overview: Given a ^31^P NMR spectrum, our model predicts the local environment around the phosphorus atom.

## Methods

### Data Sources

All experimental ^31^P NMR spectra were sourced from the Ilm-NMR-P31 data set published by Hack et al.^21^ This data set was created by digitizing existing ^31^P NMR tables.^22,23^ While information about the phosphor shift is present, for most data points the solvent, temperature, and referencing used during measurement are not provided.

### Predicting ^31^P NMR Shifts

To predict the ^31^P NMR shift, we followed the same procedure outlined by Hack et al., training a graph neural network to predict shifts based on the molecular structure. For this task, we used ChemProp, tuning the model for the best performance. The results of this optimization are shown in “Hyperparameter tuning of the ^31^P-NMR prediction model” in the Supporting Information.

For pretraining, we generated a data set by sampling a total of 665.844 phosphor-containing molecules from PubChem. Molecules were selected based on the following criteria: they contained only carbon, hydrogen, oxygen, nitrogen, phosphorus, sulfur, boron, silicon, and the halogens and their heavy-atom count (count of all atoms except for hydrogen) fell within the range of 5 to 35. The tuned ChemProp model was then used to predict the ^31^P NMR shifts for all molecules.

### Model

#### Tokenization

Chemical formulas were tokenized by splitting them into their constituent elements and occurrences. For the ^31^P NMR shifts, we employed two strategies: tokenizing the numbers by rounding each shift to one decimal place and treating each individual shift as a token or employing a multilayer perceptron (MLP) to project the shift into the embedding domain. The SMILES representation of the local phosphorus environment was tokenized using the following regular expression:
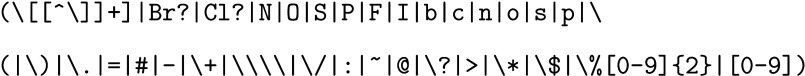


### Model Parameters

For all experiments, we employed an encoder–decoder transformer model based on the BART architecture. The model was implemented using PyTorchLightning and HuggingFace.^24,25^ The following hyperparameters were used for training:
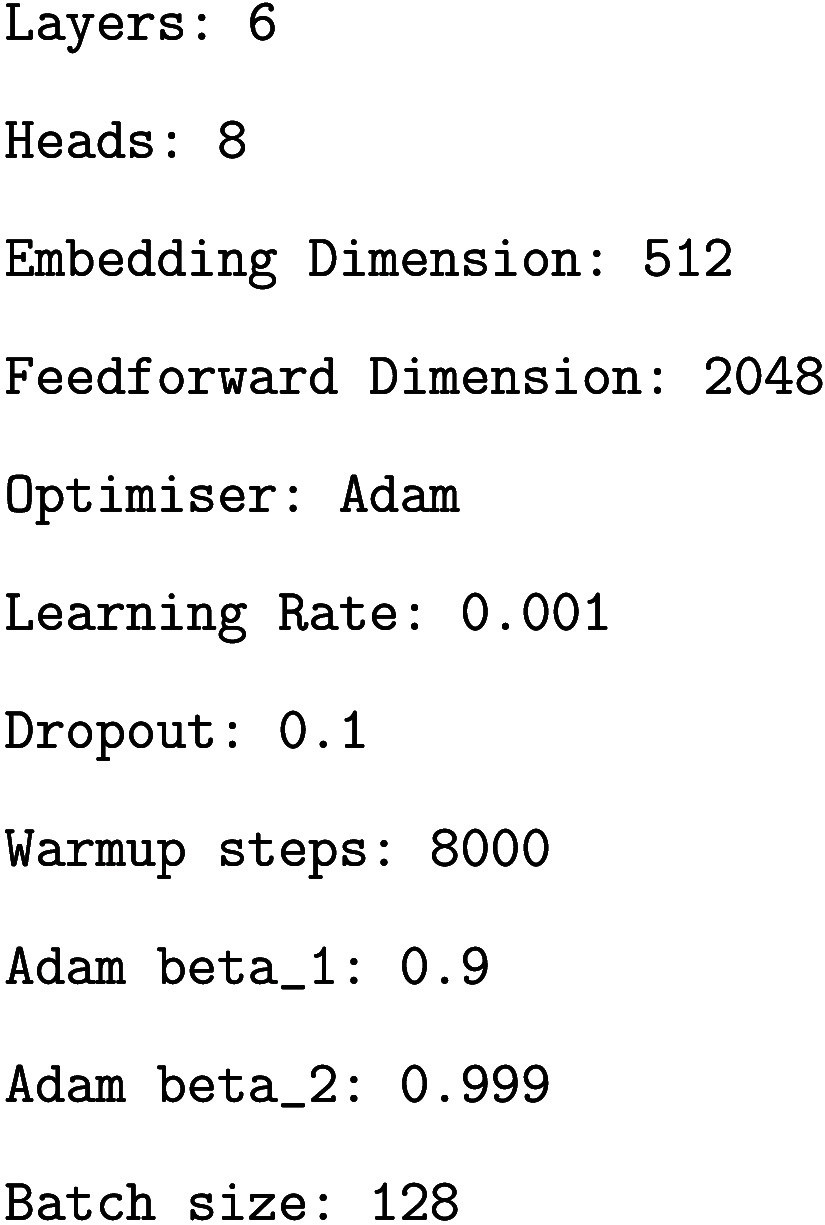


### Experimental NMRs

#### Chemicals

Cyclopentyl­triphenyl­phosphonium bromide, triphenylphosphinoxid, parathion, phosphazene base, and 4-methyl­umbelliferyl­phosphate were purchased from Sigma-Aldrich; methyltriphenyl­phosphonium bromide, 2-chlorophenyl­phosphodichloridate, and 4-chlorophenyl dichlorophosphate were purchased from Tokyo Chemical Industries; triphenylphosphine was purchased from Carl Roth GmbH; and triphenylphosphine hydrobromide was purchased from Fluorochem.

#### Procedure

Approximately 50 mg of each compound was dissolved in either deuterated chloroform or DMSO, and the solutions were transferred to 5 mm NMR tubes for analysis. ^31^P NMR spectra were recorded on a Bruker AV2-402 (400 MHz) at room temperature. Chemical shifts are expressed in parts per million (ppm) and are calibrated using residual protic solvent as the internal reference.

## Results and Discussion

### ^31^P NMR Data

In organic chemistry, ^31^P NMR spectroscopy is used to analyze the local environment around a phosphorus atom. To streamline the spectral interpretation of ^1^H-decoupled ^31^P NMR spectra (for an example, see Figure 2B), we trained a transformer model^26^ to predict the local environment of phosphorus atoms. The model was trained using the data set published by Hack et al.,^21^ which we refined to include only compounds with a heavy-atom count (non-hydrogen atoms) between 5 and 35, yielding a data set of 12,542 compounds. Each molecule in the data set contains a single phosphorus atom, with measured chemical shifts ranging from −450 to +800 ppm, 85% of which fall within the range of −100 to +100 ppm (Figure 2B).

**Figure 2:**
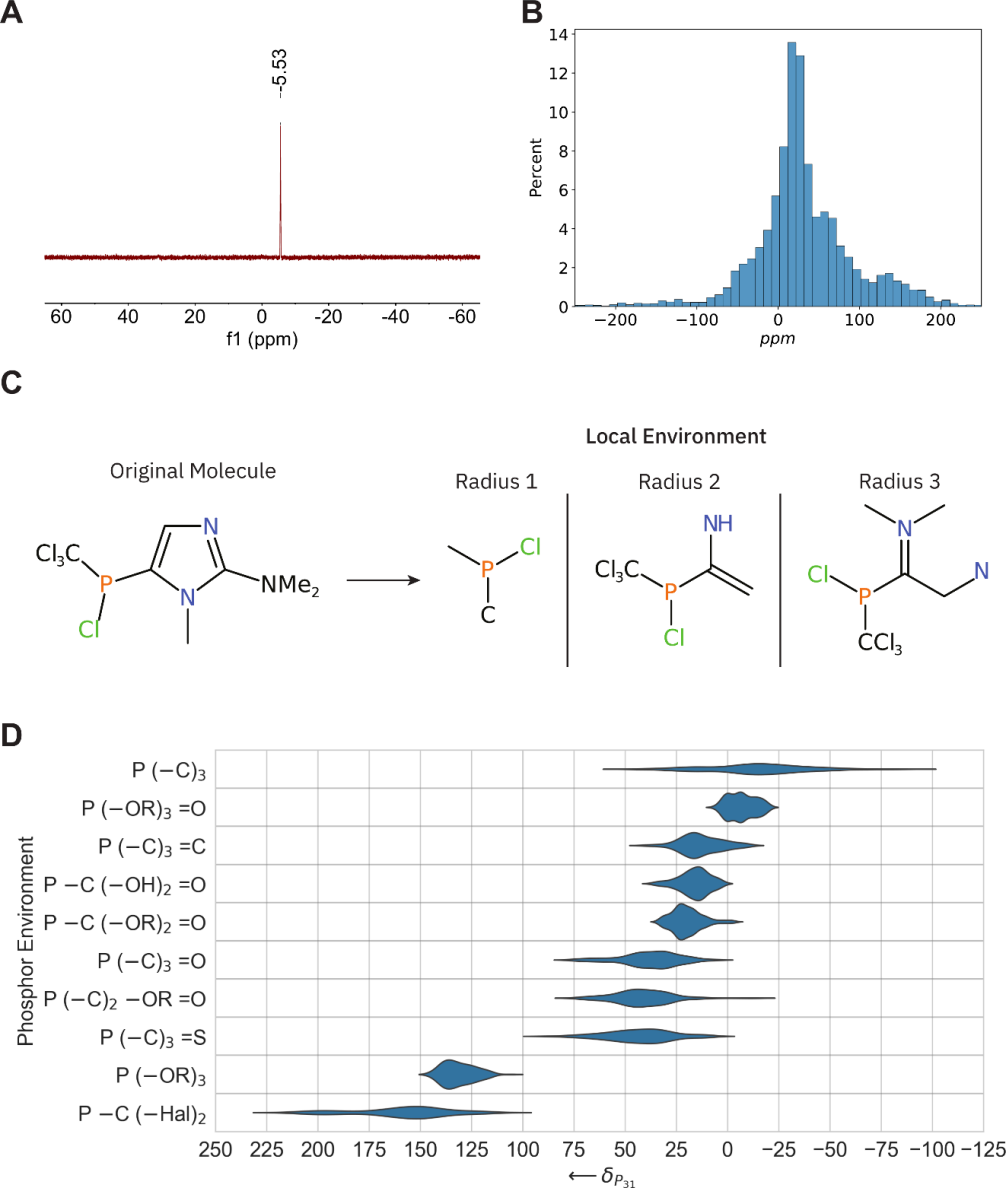
(A) A typical ^31^P NMR spectrum. We extracted the position of the peak in ppm and fed it into the model. (B) The distribution of ^31^P chemical shifts observed in the experimental data set. (C) Construction of the local environment is from the molecule. (D) Chemical shift distribution for the ten most common environments found in the data set.

While 12,542 experimental spectra represent a substantial data set in the context of experimental chemistry, this sample size is relatively small for modern machine learning applications, which typically require data sets on the scale of hundreds of thousands or even millions of samples. To overcome this limitation, we supplemented the experimental data set with additional synthetic ^31^P NMR spectra. Building on the work of Hack et al.,^21^ who demonstrated that graph neural networks (GNNs) achieve state-of-the-art performance in predicting ^31^P NMR chemical shifts, we leveraged the ChemProp library^27^ to train a GNN model capable of predicting ^31^P chemical shifts from molecular structures. This strategy enabled the generation of an additional 665,844 synthetic spectra sampled from an equal number of molecules sampled from PubChem.^28^ Full details of the prediction procedure and hyperparameters are provided in sectionPredicting ^31^P NMR Shifts.

Defining the local environment surrounding the phosphorus atom is an essential step. We characterize this environment based on the number of bonds that separate the phosphorus atom from other atoms in the molecule. All atoms within a specified bond radius are included in the local environment, with three different radii examined: one, two, and three bonds (Figure 2C). To systematically construct these environments, we used RDKit,^29^ a widely known chemoinformatics toolkit. For a bond radius of 1, this approach identified 1,060 unique local environments across the experimental data set, providing a structured representation of the local phosphorus environments for model training. To provide a more in-depth understanding of the data and the chemical shift range associated with each environment, we plot the distribution of the chemical shifts for the 10 environments with the highest occurrence in Figure 2D. A full table including all environments occurring more than 30 times can be found in SI section “^31^P NMR Reference Tables”.

### Model

To predict the local environment of phosphorus atoms, we employ an encoder–decoder transformer model using either the ^31^P chemical shift alone or in conjunction with the chemical formula. Transformer models have proven highly effective in various chemical applications, including chemical reaction predictions,^10,30,31^ automated retrosynthesis planning,^11,12^ molecular design,^32,33^ and structure elucidation of organic compounds from IR and NMR spectra.^18,19,34,35^ Our model processes a ^31^P NMR shift optionally with the chemical formula and predicts based on these inputs the local environment around the phosphorus atom. We incorporate the chemical formula alongside the chemical shift as it provides a strong prior, improving the model’s predictions by constraining the possible environments. In practical laboratory settings, the chemical formula is often readily available through mass spectrometry-based methods (e.g., LC-MS and GC-MS). Figure 3A provides an overview of our approach.

**Figure 3:**
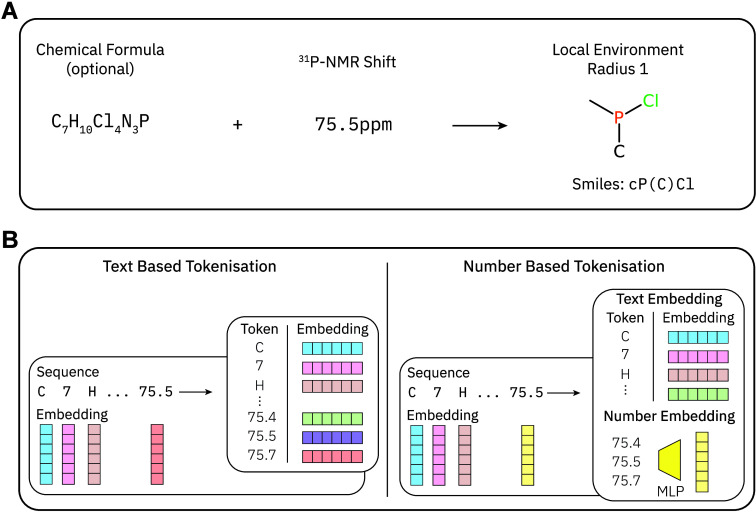
(A) Task posed to the model: Given a chemical formula and a ^31^P NMR shift, the local environment around the phosphorus atom. (B) The two different representations we explore. On the left, for text-based tokenization each number is binned to one token. On the right, our number-based tokenization approach. We project each number using an MLP into the embedding space preserving the continuous nature of numbers.

Transformer models typically consume text that is split into discrete tokens. An n-dimensional embedding vector is assigned to each token with the values of the vector learned during training. Chemical formulas are inherently textual information and are tokenized accordingly. As an example, the chemical formula C_6_H_14_ would be converted to tokens *C*, *6*, *H*, and 14. Similarly we can represent the local environment in a textual form by converting it to SMILES strings^36^ and tokenizing the strings according to Schwaller et al.^10^

However, the encoding of the ^31^P NMR chemical shift is more challenging, as it consists of continuous numerical values. Tokenizing the shifts similarly to how the chemical formula or the SMILES string are tokenized would require the use of a different embedding vector for each chemical shift value and thus would lead to a practically infinite number of vectors.

To address this issue, we explored two encoding strategies. The first approach discretizes the chemical shifts into a finite set of tokens by binning the values, effectively rounding to the first significant digit (labeled “Text Based Tokenisation” in Figure 3B and “Text” in Table 1). However, this approach lacks an inductive bias for numbers and cannot interpolate effectively, meaning that the model does not inherently recognize that, for example, 20.3 ppm is closer to 21.0 ppm than to 25 ppm. The second approach uses continuous embeddings, with a multilayer perceptron (MLP) projecting the chemical shift value directly into an embedding vector, preserving its continuous nature and enabling better interpolation (labeled “Number Based Tokenisation” in Figure 3B and “Number” in Table 1). We previously applied this method successfully to mathematical reasoning tasks.^37^

**Table 1:** Ablations of Different Encoding Procedures Including Text- and Number-Based Representation Techniques

	^31^P-Env. Radius	MLP Layers	Top-1 *↑*	Top-2 *↑*	Top-5 *↑*
Formula + Text	1	N.A.	60.27	75.41	88.47
Formula + Number	1	1	60.63	75.72	88.44
Formula + Number	1	2	**61.16**	**76.15**	**88.89**
Formula + Number	1	3	59.56	74.75	87.68

We evaluated both encoding approaches using data generated from the GNN (Table 1). The models produce five ranked predictions for each spectrum, and we assess their performance using Top-N metrics (Top-1, Top-2, and Top-5), meaning that the exact local environment is generated within the Nth prediction. We also tested MLPs with different depths, ranging from one to three layers. All approaches demonstrated similar overall performance, with MLP-based continuous embeddings slightly outperforming the categorical tokenization method. In all following experiments, we adopted an MLP with two layers as the standard encoding method. Further details on the transformer architecture and hyperparameters are provided in the method section Model Parameters (see Data Availability for the code).

### Model Evaluation

With an optimized encoding scheme for ^31^P spectra in hand, we evaluated the performance of our model when it was trained on experimental data. We explored two training strategies: (1) training models from scratch using only experimental ^31^P spectra and (2) pretraining models on generated spectra before fine-tuning them with experimental data. In total, we trained 12 different models, each evaluated using 5-fold cross-validation. The results for models trained from scratch are presented in Table 2, while those for the pretrained and fine-tuned models are shown in Table 3.

**Table 2:** Ablations on Training the Model from Scratch on the Data Provided by Hack et al.^a^

	^31^P-Env. Radius	MLP–Layers	Top-1 *↑*	Top-2 *↑*	Top-5 *↑*
Formula + Number	1	2	**52.01** ± **1.48**	**64.42** ± **1.62**	**75.97** ± **1.14**
Formula + Number	2	2	30.14 ± 1.63	37.84 ± 1.85	47.70 ± 0.65
Formula + Number	3	2	17.07 ± 1.63	22.73 ± 1.83	29.23 ± 1.58
Number	1	2	**18.73** ± **0.71**	**24.19** ± **0.98**	**33.86** ± **1.46**
Number	2	2	2.65 ± 0.93	4.43 ± 0.94	7.67 ± 1.01
Number	3	2	0.41 ± 0.15	0.78 ± 0.30	1.61 ± 0.64

a We assessed the performance across different local environment radii and whether the chemical formula is provided.

**Table 3:** Results for Models First Pretrained on the Synthetic ^31^P NMR Data and Then Fine-Tuned on the Experimental Data Set^a^

	^31^P-Env. Radius	MLP–Layers	Top-1 *↑*	Top-2 *↑*	Top-5 *↑*
Formula + Number	1	2	**53.64** ± **1.09**	**65.67** ± **0.72**	**77.69** ± **0.38**
Formula + Number	2	2	42.00 ± 1.28	52.15 ± 1.59	63.21 ± 1.53
Formula + Number	3	2	30.13 ± 1.43	39.61 ± 1.41	50.23 ± 1.49
Number	1	2	**20.43** ± **0.70**	**26.70** ± **0.89**	**36.72** ± **1.47**
Number	2	2	3.01 ± 1.02	5.75 ± 0.41	9.95 ± 0.98
Number	3	2	0.97 ± 0.50	1.57 ± 0.62	2.69 ± 0.55

a Ablations for the phosphorus environment radius and the chemical formula are provided.

Across all experiments, the model accuracy decreased as the radius of the local environment increased, reflecting the growing complexity of phosphorus coordination environments. Pretraining on generated spectra consistently improved model performance, with this effect becoming more pronounced at larger radii. While the performance difference between the two training strategies was approximately 2% for a one-bond radius, it widened to nearly 14% at a three-bond radius when the chemical formula was included. This improvement is likely due to the pretraining phase, which exposes the model to a more diverse set of chemical structures, enhancing its ability to generalize.

It is worth noting that models trained without the chemical formula exhibited significantly lower accuracy. This result suggests that multiple ^31^P environments can share highly similar chemical shifts, making additional molecular information critical to achieve accurate differentiation.

The best-performing model, a pretrained and fine-tuned model with a one-bond local environment, achieved a Top-1 accuracy of 53.64% and a Top-5 accuracy of 77.69%, demonstrating strong predictive capabilities for structure elucidation.

### Experimental Validation

Our model has demonstrated high accuracy in predicting the local phosphorus environment within the data set published by Hack et al. However, for the model to be practically useful to chemists, two critical questions must be addressed: (i) how stable is the model across different deuterated solvents and (ii) to what extent does it outperform human chemists in spectral interpretation?

Solvent effects are well known in NMR spectroscopy, often causing chemical shift variations in peak positions that introduce noise into spectral assignments.^38^ Because solvent information was available for only a fraction of the spectra in the experimental data set, we did not explicitly include it as an input feature. To assess the model’s robustness to solvent variations, we recorded the ^31^P spectra for seven compounds in deuterated chloroform (CDCl_3_) and also for seven compounds in deuterated dimethyl sulfoxide (DMSO), two of the most commonly used NMR solvents.^39^ To avoid data leakage, the experimentally measured molecules were removed from the training set. The pretrained and fine-tuned model was then evaluated on these measured spectra using a one-bond local environment. As shown in Table 4, solvent-induced shifts of up to 4 ppm were observed, with a mean difference of 1.79 ppm. Despite this variation, the model performs similarly across both solvents, suggesting that it has effectively learned to generalize across different solvent conditions. This stability likely arises from two key factors: First, the training data set includes spectra acquired under mixed solvent conditions, as well as instrumental noise, implicitly conditioning the model to handle variations in chemical shifts. Second, the use of a chemical formula as an input feature provides a structural prior, constraining the model’s predictions to chemically reasonable shifts. Additional details on the compounds and NMR procedures are provided in Methods section Experimental NMRs.

**Table 4:** Comparison of the Performance of the Model on Spectra Measured in Deuterated Chloroform and DMSO^a^

	Solvent	N-Molecules	Radius	Top-1 *↑*	Top-2 *↑*	Top-5 *↑*
Formula + Number	CDCl_3_	7	1	60.00 ± 14.00	62.86 ± 11.43	68.57 ± 14.00
Formula + Number	DMSO-d6	7	1	57.14 ± 29.97	82.86 ± 5.71	88.57 ± 5.71
Formula + Number	CDCl_3_	7	1	60.00 ± 14.00	62.86 ± 11.43	68.57 ± 14.00
Chemists	CDCl_3_	7	1	35.71 ± 10.09	49.99 ± 10.10	49.99 ± 10.10

a The performance of our model was compared to that of human chemists.

To assess how the model compares to expert chemists, we conducted a direct performance benchmark. Two experienced chemists were given the same task as the model: using a provided chemical formula and the ^31^P NMR spectrum, they were asked to predict and rank the five most likely local phosphorus environments. They were allowed to consult standard NMR references and online databases. The results, summarized in Table 4, show that the model outperforms the human chemists by 25%, demonstrating its potential and efficiency as a tool for structure elucidation.

## Conclusions

In this study, we developed an AI model to automate the interpretation of ^31^P NMR spectra, providing chemists with a powerful tool for structure elucidation. Specifically, we introduced a transformer-based model capable of predicting the local environment around phosphorus atoms directly from ^31^P NMR spectral data with the option to incorporate the molecular formula for improved accuracy. The model was first pretrained on a large data set of generated ^31^P NMR spectra and subsequently fine-tuned using experimental data. Our best-performing model achieved a Top-1 accuracy of 53.64%, Top-2 accuracy of 65.67%, and Top-5 accuracy of 77.69%.

Additionally, we demonstrated that the model remains robust across different solvent conditions by evaluating its performance on seven spectra recorded in chloroform and seven spectra recorded in DMSO. The model is able to predict the local environment for both solvents without any loss in performance. Furthermore, the model was benchmarked against human chemist experts in spectral assignment tasks, where it demonstrated superior performance, outperforming two experienced chemists by 25%.

These results highlight the potential of AI-assisted NMR interpretation to enhance efficiency and accuracy in structure elucidation, making it a valuable resource for both research and industrial applications.

## Supplementary Material



## Data Availability

The code supporting the findings of this work is available at https://github.com/rxn4chemistry/MultimodalAnalytical. Instructions on how to replicate the results and data are provided. The synthetic ^31^P NMR data is provided openly here: https://zenodo.org/records/14971859

## Notes

Additional experiments, ^31^P-reference table, and experimental spectra (PDF)
