# Supplementary material for: From Spectra to Structure: AI-Powered 31P NMR Interpretation
Source: Anal Chem. 2025 Jul 16;97(29):15736–42. doi: 10.1021/acs.analchem.5c01460 (PMC12311896; doi:10.1021/acs.analchem.5c01460)
Supplement: Supplementary file 1 [file ac5c01460_si_001.pdf]

Supporting Information: From Spectra to Structure: AI-Powered ^{31}P-NMR Interpretation

Marvin Alberts,[*,†,‡,¶] Nina Hartrampf,[‡] and Teodoro Laino[†,¶]

†IBM Research Europe, Säumerstrasse 4, 8803 Rüschlikon, Switzerland

‡University of Zurich, Department of Chemistry, Winterthurerstrasse 190, 8057 Zurich, Switzerland

¶NCCR Catalysis, Wildhainweg 3, 3001 Bern, Switzerland

E-mail: marvin.alberts@ibm.com

Contents

1 Hyperparameter tuning of the ^{31}P-NMR prediction model S2

2 Classification model S3

3 ^{31}P-NMR Reference Tables S5

4 Experimental NMR-Spectra S9

4.1 $CDCl_3$ S9

4.2 DMSO S13

References S17

1 Hyperparameter tuning of the ^{31}P-NMR prediction model

We use Chemprop to predict ^{31}P-NMR shifts as a regression task.[1] The model is trained on the 14.210 spectra provided by Hack et. al. We tune the model's message passing dimension, the depth of the model and the learning rate. In all cases we report the mean absolute error and the root mean squared error. The results are shown below in Table S1.

With the best hyperparameter combination found, we use ensembling to improve the performance. The results for the ensembling are shown in Table S2.

Table S1: ^{31}P-NMR Prediction performance across hyperparameters.

Message Passing Dimension	Model Depth	Learning Rate	MAE ↓	RMSE ↓
400	3	0.001	**10.271**	20.678
200	3	0.001	11.150	**19.991**
200	2	0.001	11.400	21.026
300	5	0.001	10.731	21.747
400	2	0.001	11.057	21.438
100	5	0.001	11.644	20.978
300	3	0.001	11.050	21.962
300	4	0.001	11.352	21.766
300	3	0.0001	11.786	21.710
400	4	0.0001	11.869	21.681
50	5	0.001	12.260	21.298
200	5	0.001	12.022	21.597
100	2	0.001	12.216	21.430
300	2	0.0001	12.158	21.577
200	4	0.001	11.875	21.943
300	2	0.001	12.249	21.761
400	4	0.001	11.097	23.159
400	2	0.0001	12.139	22.325
50	4	0.001	12.813	21.781
300	4	0.0001	12.499	22.148
400	5	0.001	11.966	22.953
400	3	0.0001	12.273	22.657
200	3	0.0001	12.772	22.212
200	2	0.0001	12.829	22.314
50	3	0.001	13.339	21.880
100	4	0.001	12.587	22.640
50	2	0.001	13.382	21.973
100	3	0.001	12.566	22.861
400	5	0.0001	12.926	22.691
200	4	0.0001	12.971	22.867
300	5	0.0001	12.857	23.047
100	3	0.0001	13.599	23.529
100	2	0.0001	14.051	23.432
100	4	0.0001	14.217	23.787
100	5	0.0001	14.112	24.397
200	5	0.0001	14.145	24.827
50	5	0.0001	15.659	24.633
50	3	0.0001	15.792	25.453
50	4	0.0001	15.508	25.812
50	2	0.0001	15.785	25.878

2 Classification model

As there are a limited number of phosphor environments in the dataset, predicting the environment around a phosphor atom can be considered a classification task. We trained

Table S2: ^{31}P-NMR Prediction performance of ensembles of different sizes.

Ensemble	MAE ↓	RMSE ↓
5 Models	**9.130**	**19.093**
4 Models	9.467	19.455
3 Models	9.255	19.842
2 Models	9.822	19.825

an encoder-only transformer model to predict the correct phosphor environment class. We only consider environments with a radius of one bond around the central phosphorus atom as the number of unique environments increases exponentially with the radius. We select a total of 3246 phosphor environments to classify. The following hyperparameters are used for the encoder-only model:

```
Layers: 4
Heads: 8
Embedding Dimension: 512
Feedforward Dimension: 2048
Optimiser: Adam
Learning Rate: 0.001
Dropout: 0.1
Warmup steps: 8000
Adam beta_1: 0.9
Adam beta_2: 0.999
Batch size: 128
```

We train each model for 60 epochs. We assess the performance of a model pretrained on synthetic data and finetuned on experimental data in comparison to one only trained on experimental data. Results are shown in Table S3.

Table S3: ^{31}P-NMR Classification

	Chemical Formula	Radius	MLP-Layers	Accuracy (Top-1) ↑	F1-Score ↑
From Scratch	✗	1	2	18.98 ± 0.96	0.237 ± 0.013
From Scratch	✓	1	2	35.06 ± 1.09	0.408 ± 0.012
Pretrained	✗	1	2	20.51 ± 1.12	0.240 ± 0.014
Pretrained	✓	1	2	41.28 ± 1.20	0.448 ± 0.013

3 ^{31}P-NMR Reference Tables

Chemists traditionally rely on reference tables for interpreting ^{31}P-NMR spectra,[2,3] which catalogue phosphorus environments alongside their established chemical shift ranges. However, most existing tables were compiled decades ago and may not reflect the full scope of available spectroscopic data. As an alternative, we developed an updated reference based on the dataset published by Hack et al.[4].

Our analysis focused on phosphorus environments (radius 1) with sufficient representation, selecting those appearing more than 30 times. This yielded 60 unique environments covering 6,642 spectra from the complete dataset of 12,542 datapoints. To ensure the quality of the data, we excluded outliers and problematic measurements by removing all datapoints beyond the 2.5th and 97.5th percentiles.

The resulting distributions are presented in Figure S1 and Figure S2, organised from lowest to highest mean chemical shift. Unlike traditional reference tables that show only chemical shift ranges, our plot displays the distribution for each environment, providing information about both the typical shift ranges as well as the variability for each environment.

Like all reference tables, the ones presented here carry inherent limitations primarily arising from the dataset used. Although constructed from 6,642 spectra, the molecules contained in the dataset do not uniformly sample the chemical space. Consequently, certain phosphorus environments are likely overrepresented while others remain undersampled, potentially skewing the displayed environments.

Similarly, the distributions presented here reflect both the chemical reality and the sampling biases of the source dataset. For environments with limited occurrences in the dataset, the table may not accurately capture the full range of chemical shifts encountered in practice. Despite these limitations, we believe this resource offers value to the spectroscopic community, providing a more comprehensive reference than previously available while also providing information on the distribution of chemical shifts for each environment.

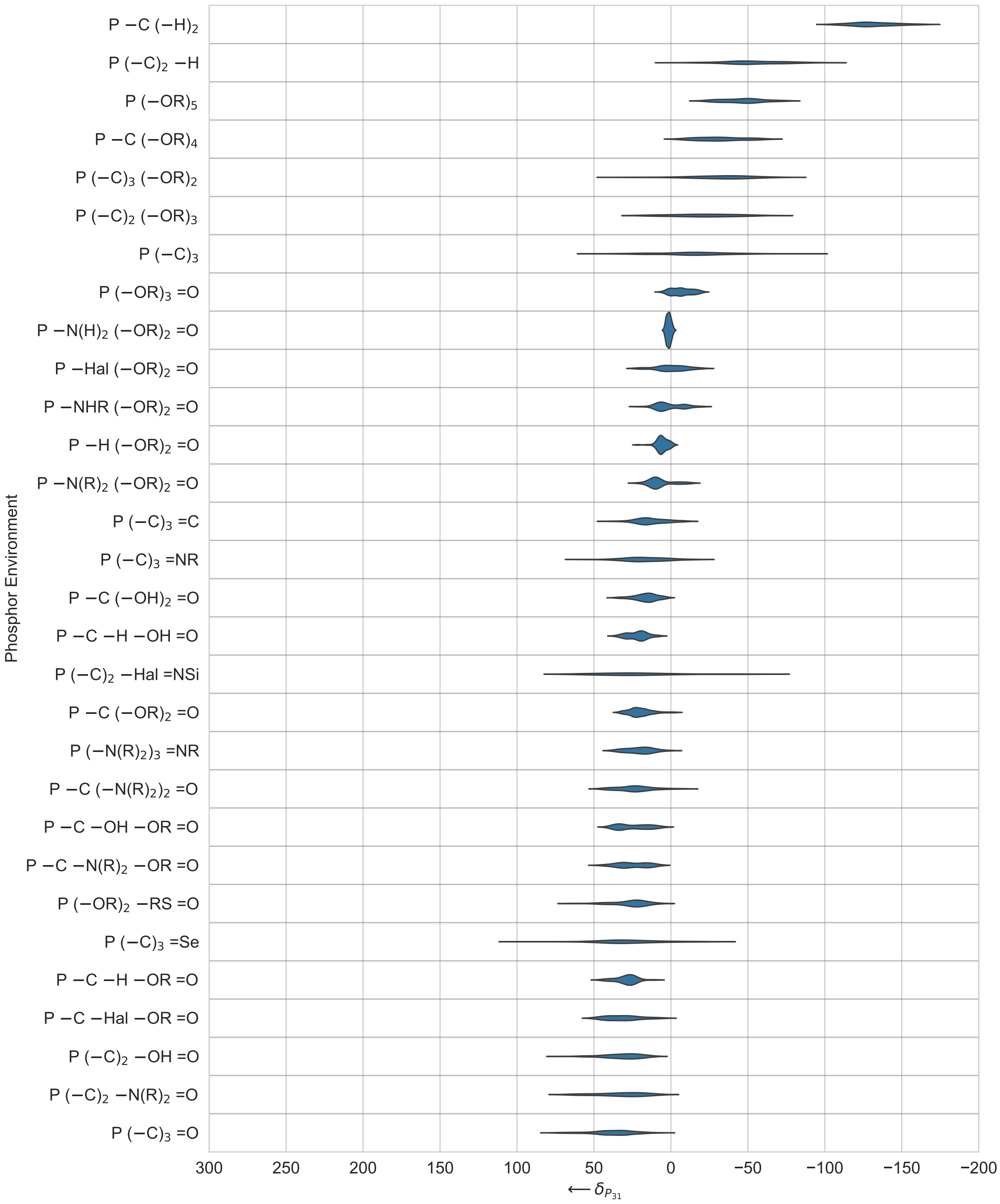
P −C (−H)2
P (−C)2 −H
P (−OR)5
P −C (−OR)4
P (−C)3 (−OR)2
P (−C)2 (−OR)3
P (−C)3
P (−OR)3 =O
P −N(H)2 (−OR)2 =O
P −Hal (−OR)2 =O
P −NHR (−OR)2 =O
P −H (−OR)2 =O
P −N(R)2 (−OR)2 =O
P (−C)3 =C
P (−C)3 =NR
P −C (−OH)2 =O
P −C −H −OH =O
P (−C)2 −Hal =NSi
P −C (−OR)2 =O
P (−N(R)2)3 =NR
P −C (−N(R)2)2 =O
P −C −OH −OR =O
P −C −N(R)2 −OR =O
P (−OR)2 −RS =O
P (−C)3 =Se
P −C −H −OR =O
P −C −Hal −OR =O
P (−C)2 −OH =O
P (−C)2 −N(R)2 =O
P (−C)3 =O
Phosphor Environment
300
250
200
150
100
50
0
−50
−100
−150
−200
← δP31

Figure S1

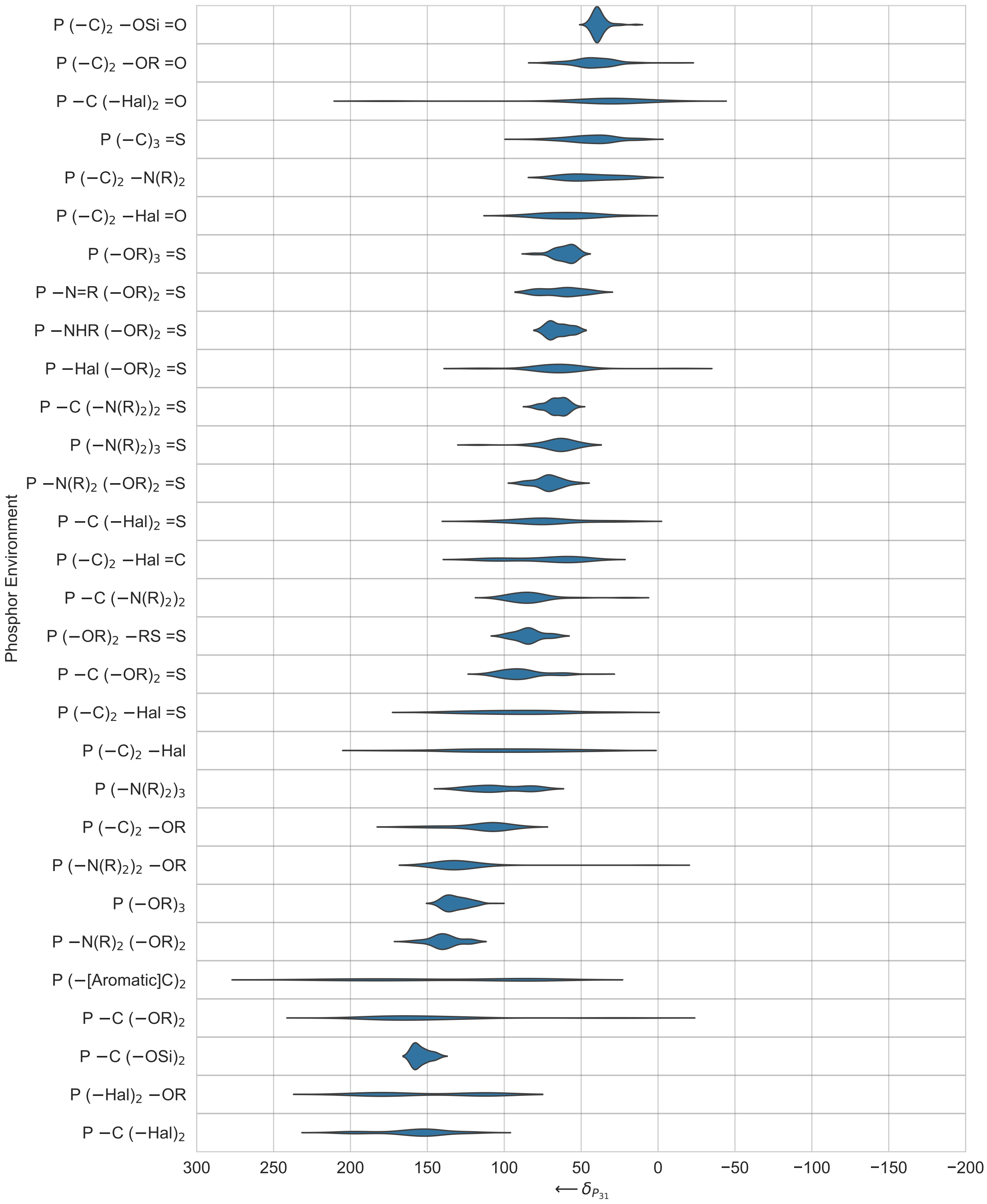

P (−C)2 −OSi =O
P (−C)2 −OR =O
P −C (−Hal)2 =O
P (−C)3 =S
P (−C)2 −N(R)2
P (−C)2 −Hal =O
P (−OR)3 =S
P −N=R (−OR)2 =S
P −NHR (−OR)2 =S
P −Hal (−OR)2 =S
P −C (−N(R)2)2 =S
P (−N(R)2)3 =S
P −N(R)2 (−OR)2 =S
P −C (−Hal)2 =S
P (−C)2 −Hal =C
P −C (−N(R)2)2
P (−OR)2 −RS =S
P −C (−OR)2 =S
P (−C)2 −Hal =S
P (−C)2 −Hal
P (−N(R)2)3
P (−C)2 −OR
P (−N(R)2)2 −OR
P (−OR)3
P −N(R)2 (−OR)2
P (−[Aromatic]C)2
P −C (−OR)2
P −C (−OSi)2
P (−Hal)2 −OR
P −C (−Hal)2
Phosphor Environment
300
250
200
150
100
50
0
−50
−100
−150
−200
⟵ $\delta_{P_{31}}$

Figure S2

4 Experimental NMR-Spectra

4.1 $CDCl_3$

Cyclopentyltriphenylphosphonium bromide – $CDCl_3$

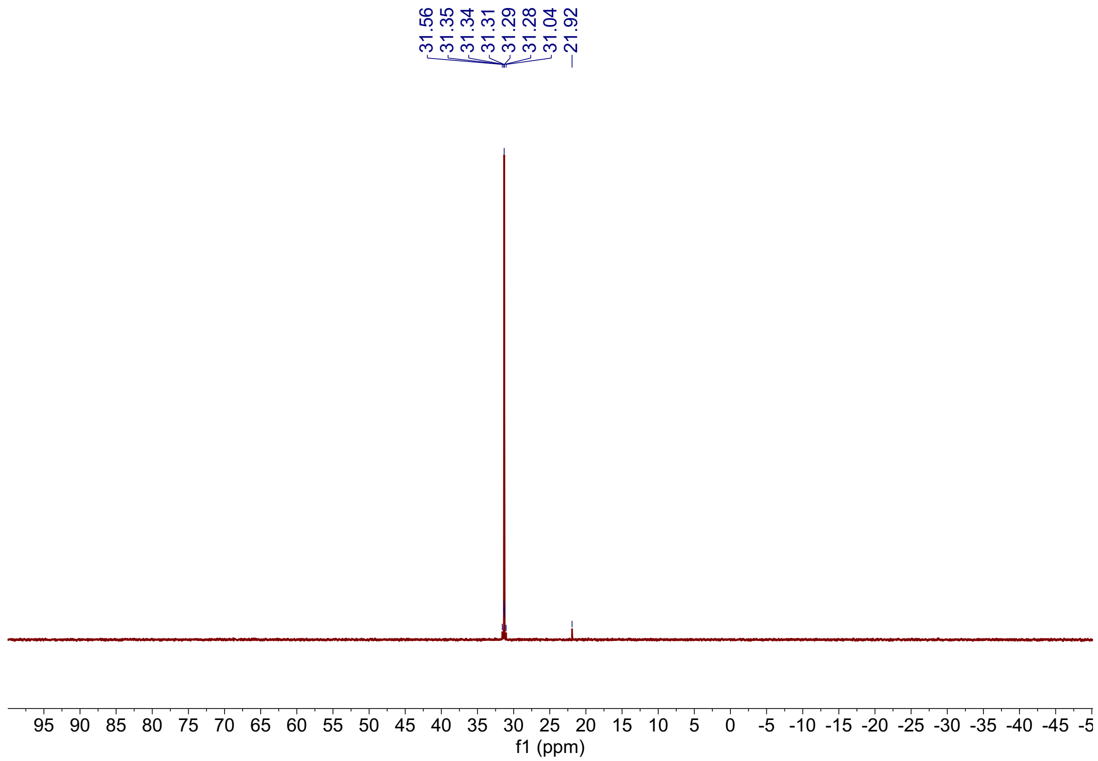

Triphenylphosphinoxid – $CDCl_3$

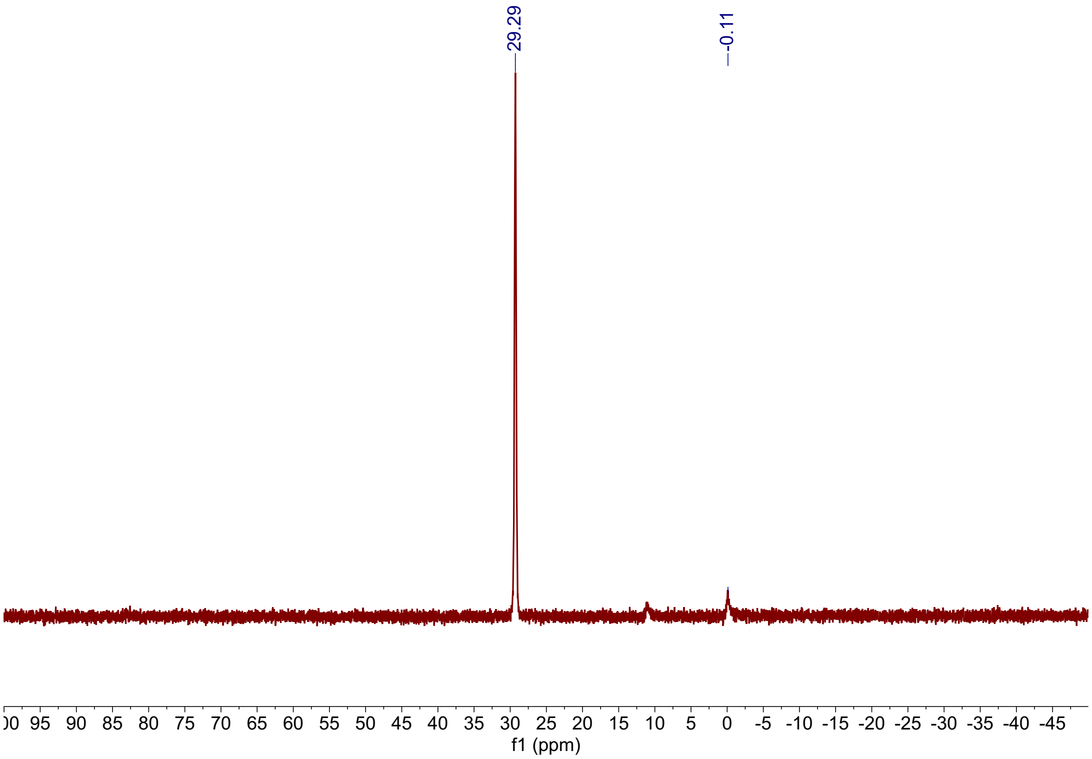

Parathion – $CDCl_3$

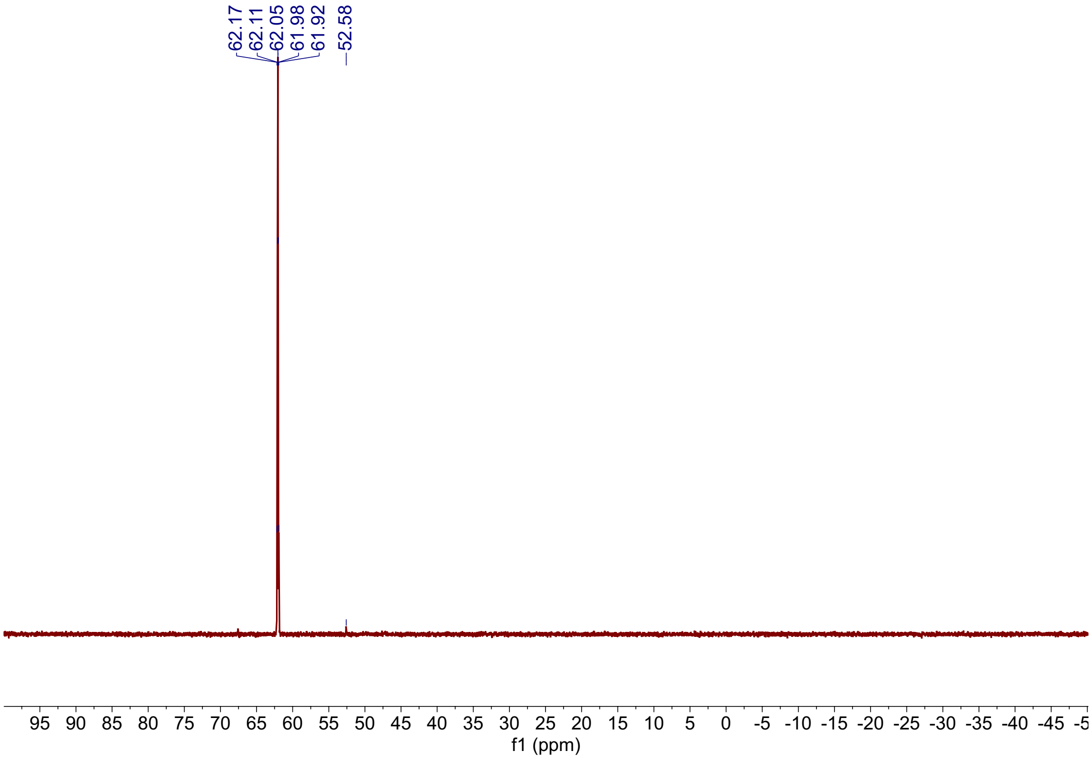

2-Chlorophenylphosphodichloridate – $CDCl_3$

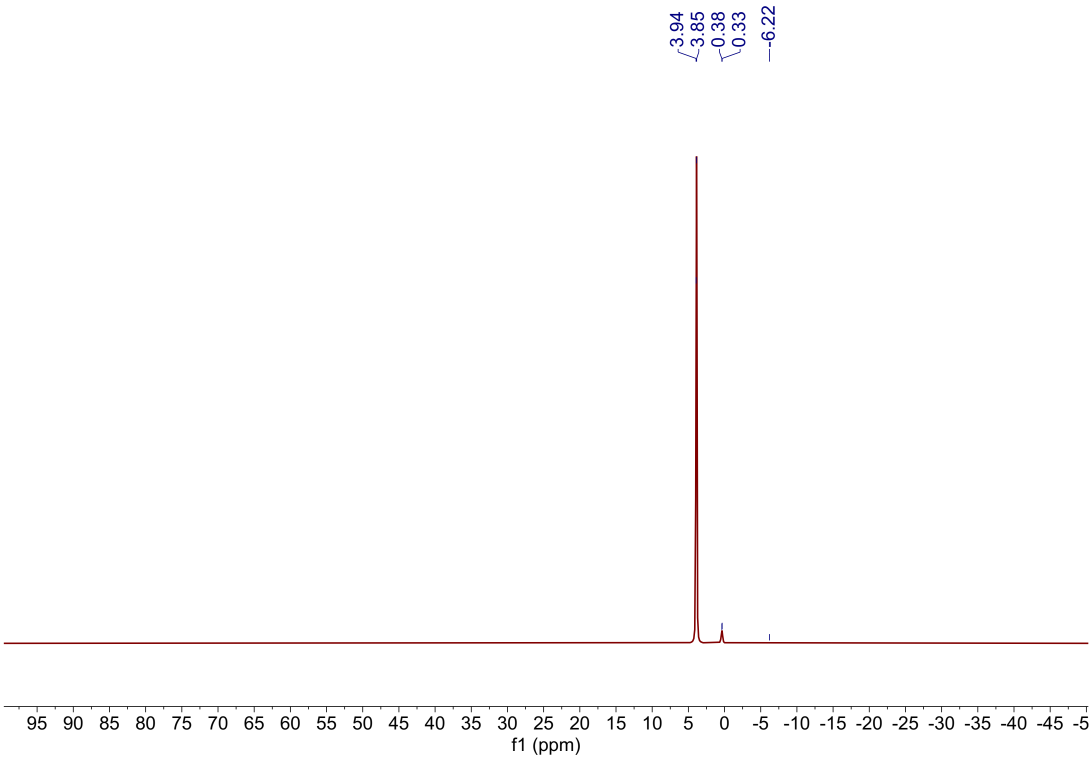

Triphenylphosphine – $CDCl_3$

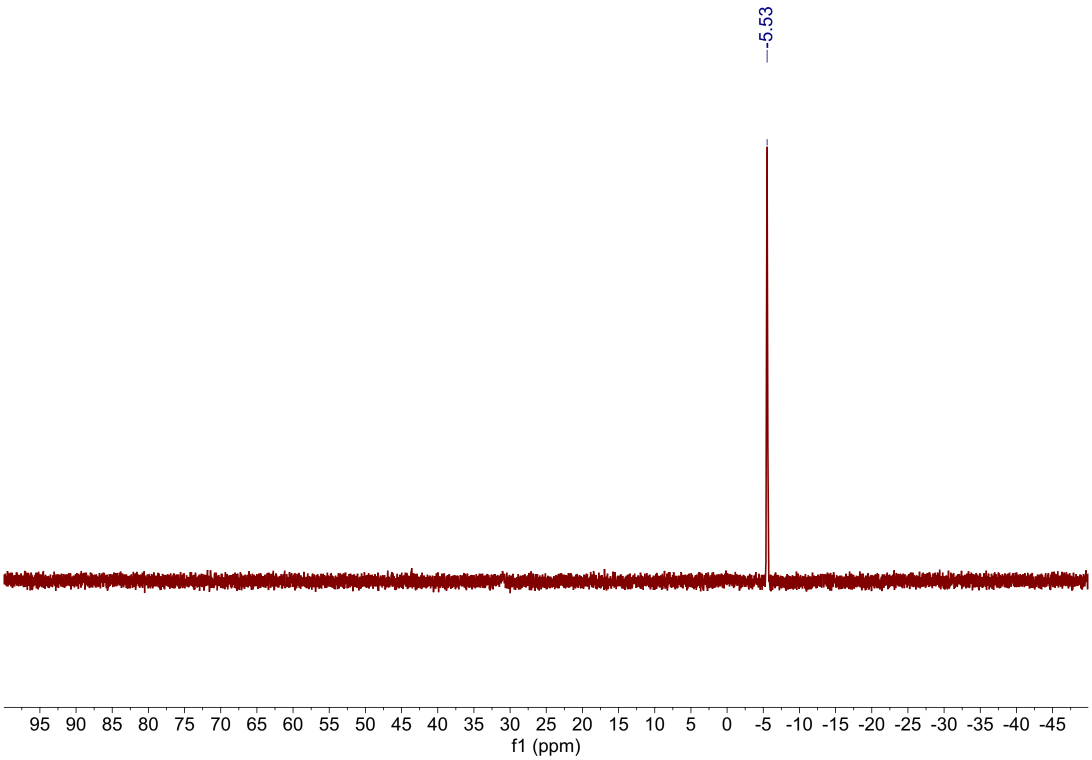

4-Chlorophenyl dichlorophosphate – $CDCl_3$

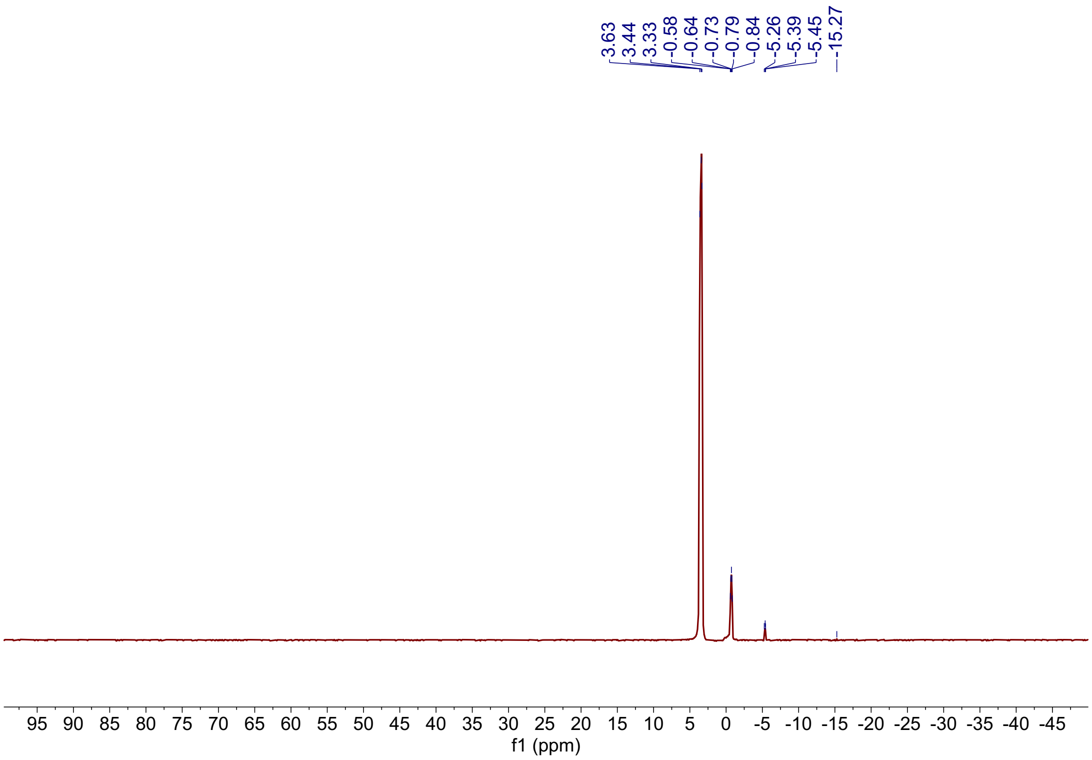

Triphenylphosphine hydrobromide – $CDCl_3$

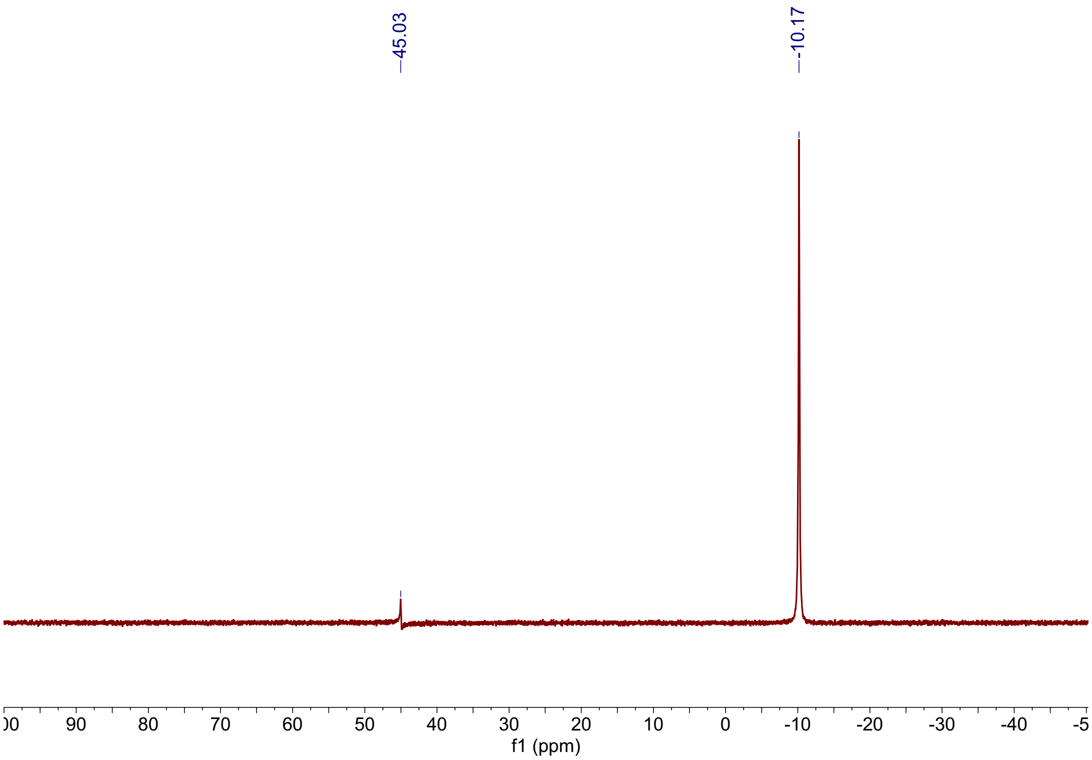

4.2 DMSO

Cyclopentyltriphenylphosphonium bromide – DMSO

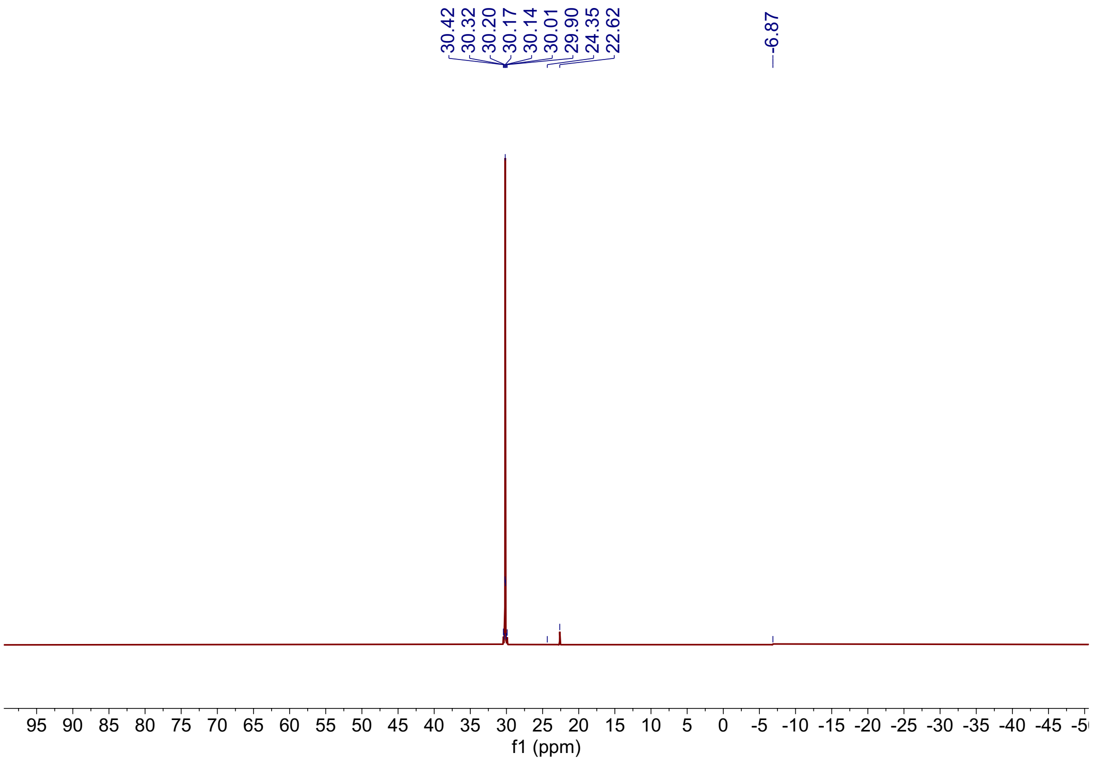

Triphenylphosphinoxid – DMSO

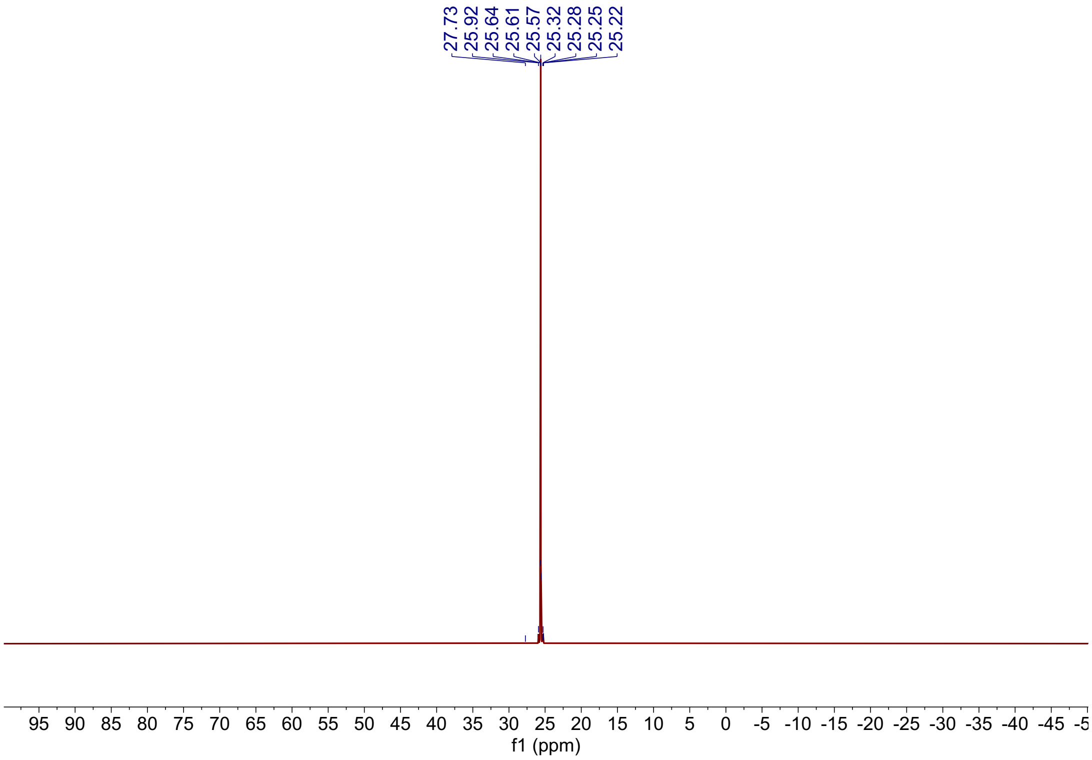

Methyltriphenylphosphonium bromide – DMSO

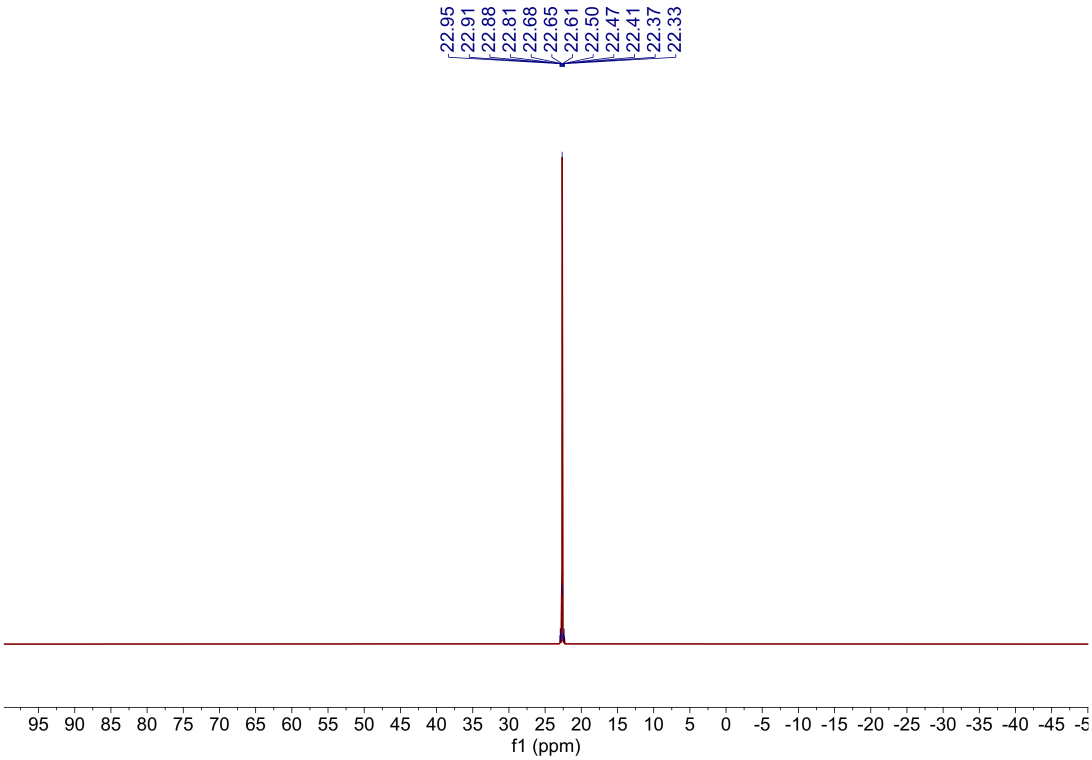

Parathion – DMSO

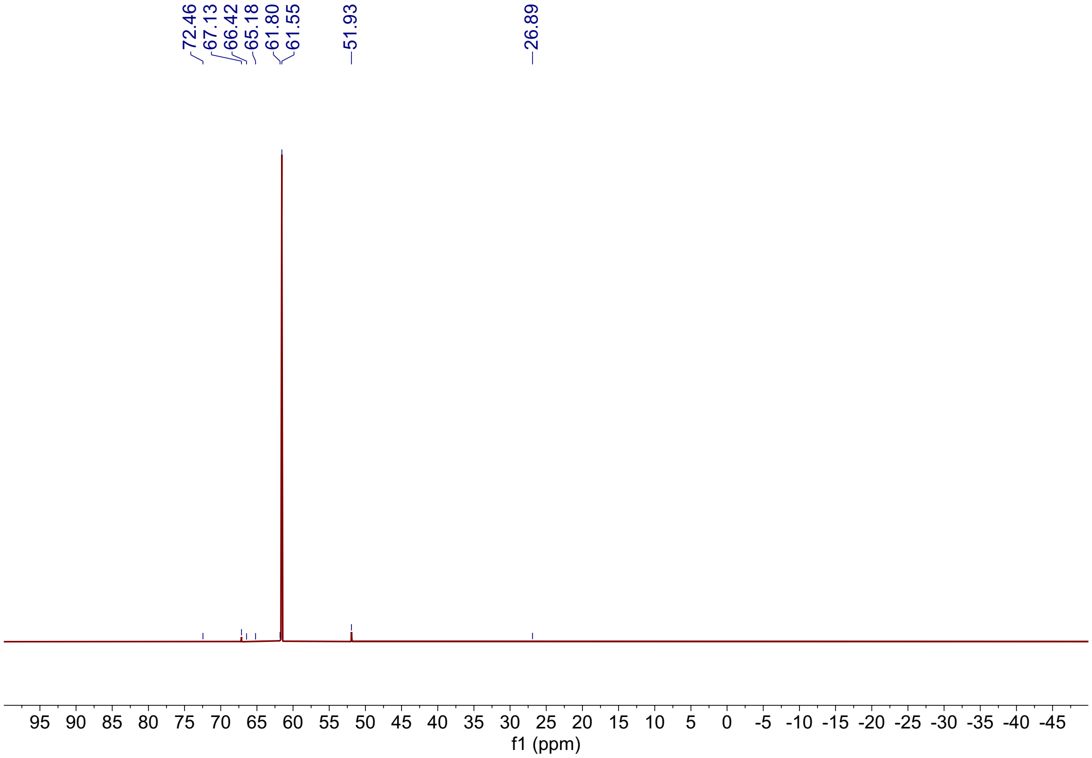

Triphenylphosphine – DMSO

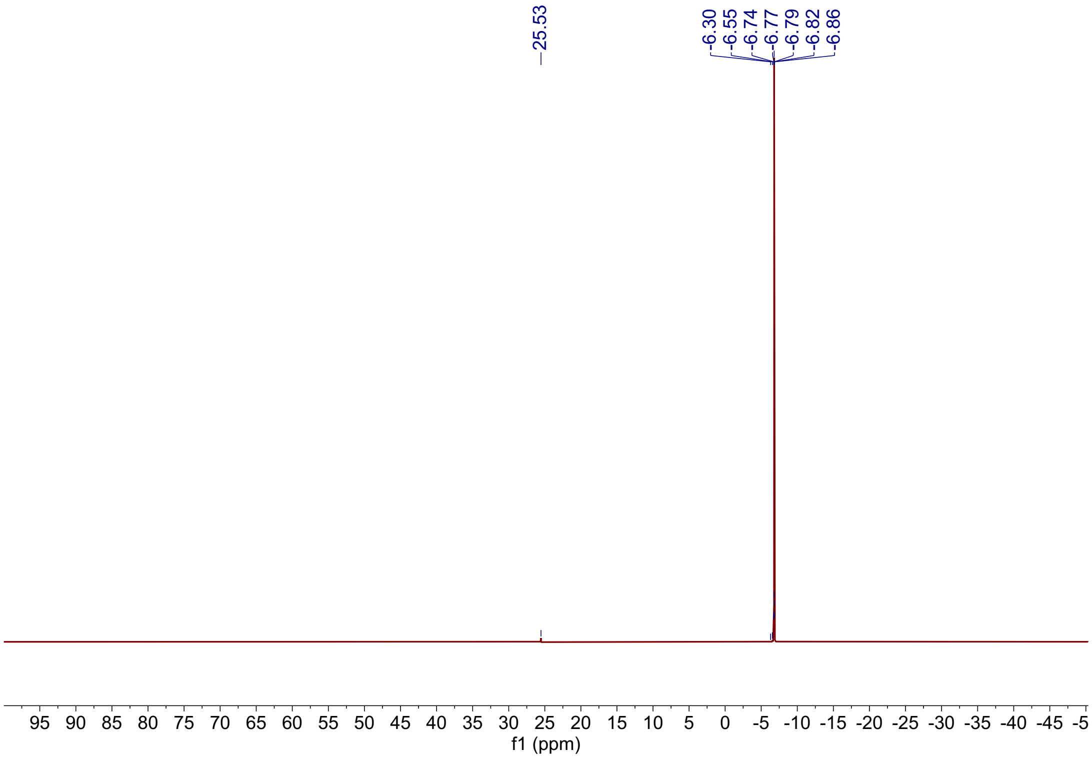

Phosphazene Base – DMSO

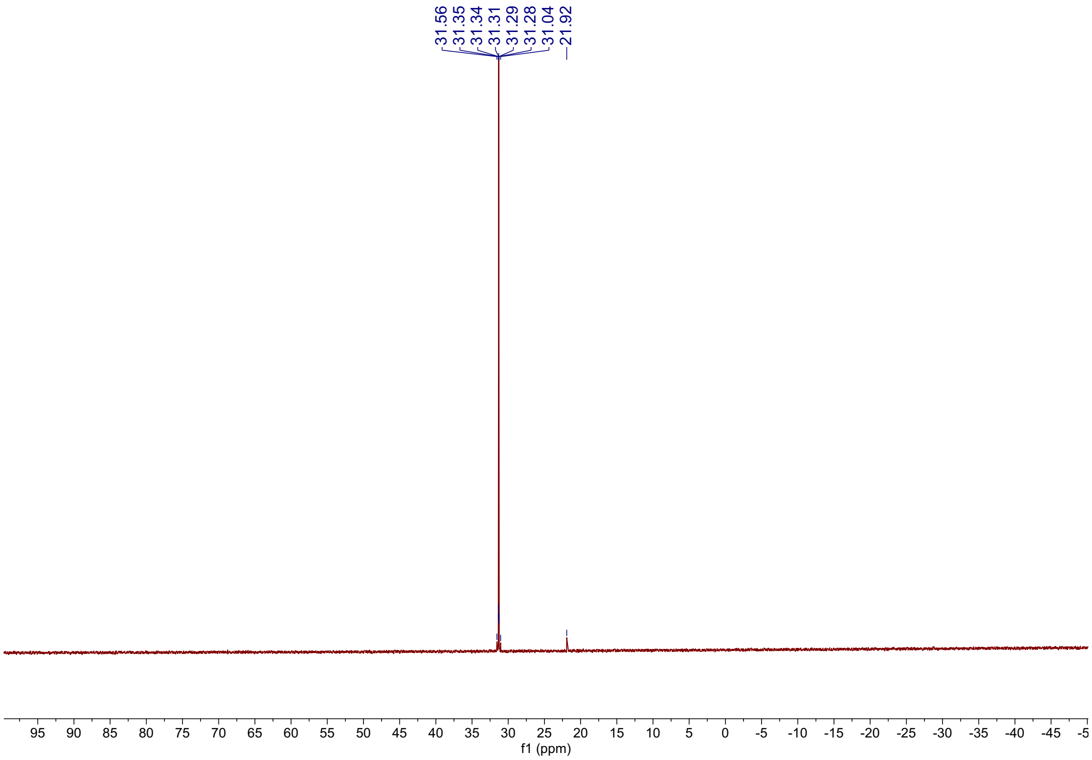

4-Methylumbelliferylphosphate – DMSO

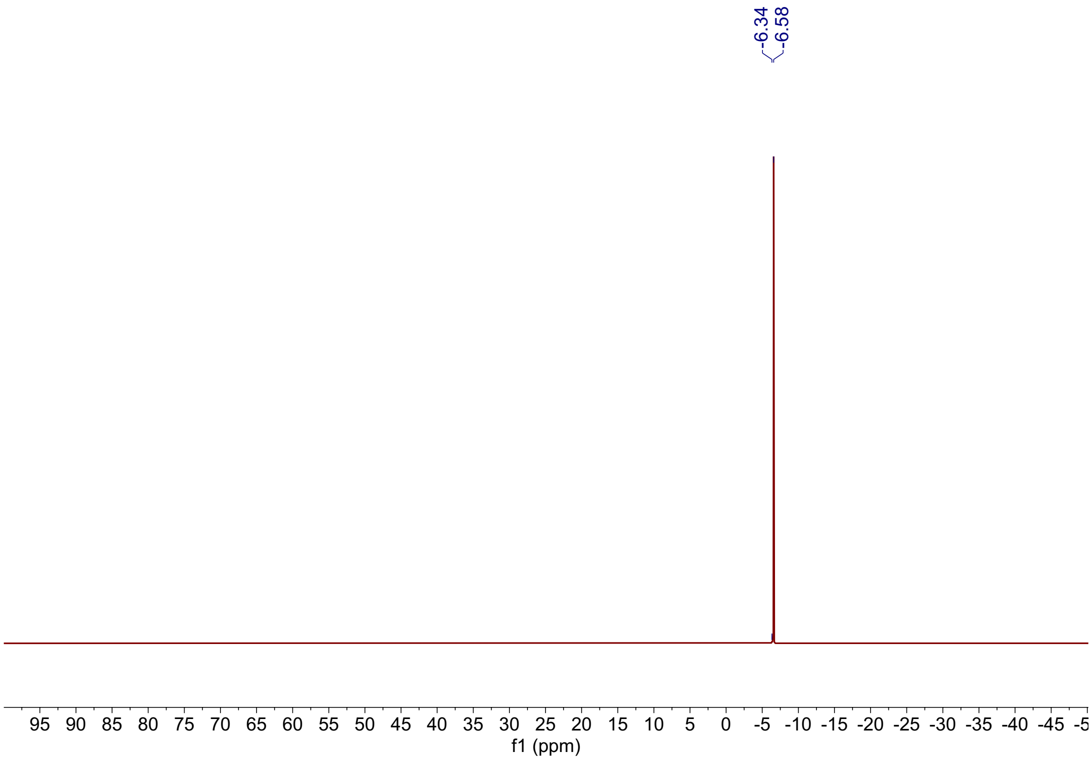

Triphenylphosphine hydrobromide – DMSO

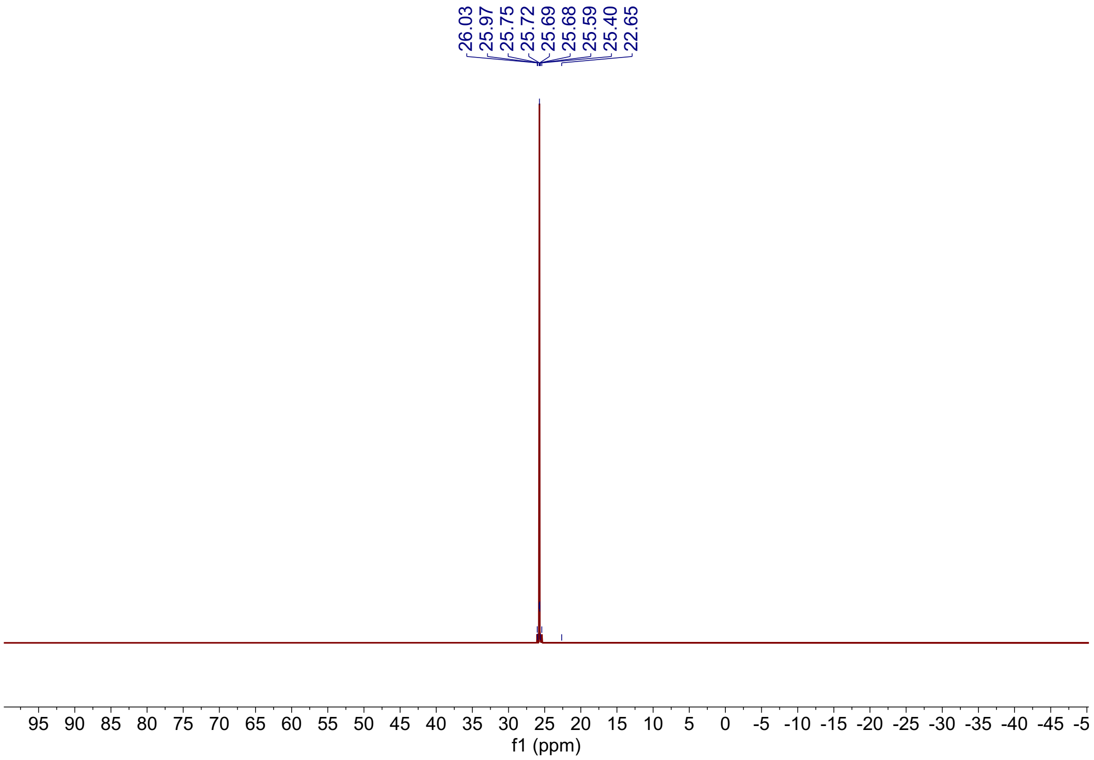

References

(1) Heid, E.; Greenman, K. P.; Chung, Y.; Li, S.-C.; Graff, D. E.; Vermeire, F. H.; Wu, H.; Green, W. H.; McGill, C. J. Chemprop: A Machine Learning Package for Chemical Property Prediction | Journal of Chemical Information and Modeling. *Journal of Chemical Information and Modeling* **2024**, *64*, 9–17.

(2) Berger, S.; Braun, S.; Kalinowski, H.-O. ^{31}P-NMR-Spektroskopie. 1993.

(3) Kühl, O., Ed. *Phosphorus-31 NMR Spectroscopy*; Springer: Berlin, Heidelberg, 2009.

(4) Hack, J.; Jordan, M.; Schmitt, A.; Raru, M.; Zorn, H. S.; Seyfarth, A.; Eulenberger, I.; Geitner, R. Ilm-NMR-P31: an open-access 31P nuclear magnetic resonance database and data-driven prediction of 31P NMR shifts. *Journal of Cheminformatics* **2023**, *15*, 122.
